# For Hæmorrhage

**Published:** 1891-09

**Authors:** 


FOR HÆMORRHAGE.—Dr. Ferguson recommends in those cases where there is a strong tendency to hæmorrhage ten or fifteen drops every two or three hours of a mixture composed of one ounce each of alcohol and oil of turpentine, to which is slowly added one ounce of sulphuric acid.—*Med. Record.*—*Southern Clinic.*

